# IMP1 suppresses breast tumor growth and metastasis through the regulation of its target mRNAs

**DOI:** 10.18632/oncotarget.7464

**Published:** 2016-02-17

**Authors:** Guangli Wang, Zhenqiang Huang, Xin Liu, Wenhe Huang, Shaoying Chen, Yanchun Zhou, Deling Li, Robert H. Singer, Wei Gu

**Affiliations:** ^1^ Department of Pathophysiology, The Key Immunopathology Laboratory of Guangdong Province, Shantou University Medical College, Shantou, Guangdong Province, 515031, China; ^2^ Tumor Hospital, Shantou University Medical College, Shantou, Guangdong Province, 515031, China; ^3^ Department of Anatomy and Structural Biology, Albert Einstein College of Medicine, Bronx, NY 10461, USA

**Keywords:** breast cancer growth and metastasis, gene expression, post-transcriptional regulation of mRNAs, RNA-binding protein, ZBP1/IMP1

## Abstract

We have previously reported the ability of IMP1 in inhibiting proliferation and invasiveness of breast carcinoma cells *in vitro*. In the current study, we utilized a mouse xenograft model to further investigate the function of IMP1 in breast tumor progression and its underlying mechanism. We demonstrated that IMP1 expression significantly suppressed the growth of MDA231 cell-derived xenograft tumors and subsequent lung metastasis. Microarray analyses and differential gene expression identified handful mRNAs, many of which were involved in breast tumor-growth and metastasis. Further studies revealed that these mRNAs were directly interacted with the KH34 domain of IMP1 and this interaction post-transcriptionally regulated their corresponding protein expression. Either deletion of the KH34 domain of IMP1 or alteration of the expression of IMP1-bound mRNAs affected cell proliferation and tumor growth, producing the same phenotypes as IMP1 knockdown. Correlation of increased IMP1 expression with the reduced levels of its bound mRNAs, such as PTGS2, GDF15 and IGF-2 transcripts, was also observed in human breast tumors. Our studies provide insights into a molecular mechanism that the positive function of IMP1 to inhibit breast tumor growth and metastasis could be through the regulation of its target mRNAs.

## INTRODUCTION

Breast cancer is the most common cancer diagnosed and the second leading cause of cancer deaths in women [1]. In the United States alone, approximately 40,000 women die of breast cancer each year, largely attributed to systemic metastasis [2, 3]. Therefore, identifying factors that associate with suppressing breast cancer aggressiveness and metastasis would have the potential to serve as novel molecular targets for breast cancer therapy.

IMP1/ZBP1 (hereafter referred to as IMP1) has been implicated in many aspects of RNA regulation [4]. In a variety of cell types, IMP1 regulates the localization of β-actin mRNA, resulting in the asymmetric translation of β-actin protein and enhances cell polarity [5]. In mice, binding of the IMP1 orthologue (CRD-BP) to the coding region of c-myc mRNA increases it stability [6]. The human IMP1 was originally identified as a translational repressor of mRNA encoding insulin-like growth factor 2 (IGF-2), but has since been found to involve in the localization of many other mRNAs, including H19, tau, CD44, β-catenin and E-cadherin mRNAs [4, 7]. Local translation of β-actin, CD44 or E-cadherin mRNA mediated by IMP1 has been shown to involve in actin dynamics, invadopodia formation and cell-cell adhesions. Loss of IMP1 function deregulates mRNAs normally associated with the protein and alters many important cellular processes, such as cell polarity and migration [7–9].

The biological consequence of IMP1 expression in tumorigenesis and metastasis is remained elusive. Active expression of the IMP1 gene has been observed in human breast (58%), ovarian, and colorectal (81%) tumors [10, 11]. IMP1 knockdown reduces proliferation and survival of ovarian cancer cells [12]. Targeted overexpression of IMP1 in mammary tissues of transgenic mice induced breast tumor [13]. However, studies have also indicated a suppressive function of IMP1 in proliferation and invasiveness of breast carcinoma cells. In IMP1 non-expressing cells, re-expression of IMP1 reduced proliferation and invasive potential of metastatic cells [7, 9, 14, 15]. In a rat xenograft study, Wang et al. found that expression of IMP1 in an IMP1-negative metastatic MTLn3 line inhibited lung metastasis of the cell-derived breast tumors [16]. More interestingly, although IMP1 was considered as an oncogene for the colorectal tumor growth and as a potential initiator of metastasis [17], a recent study reported that loss of IMP1 function in stromal cell provided a microenvironment that promoted colon tumorigenesis [18].

In this study, we utilized orthotopic breast fat pad xenografts to further investigate the functions of IMP1 in human breast tumorigenesis and metastasis *in vivo*. We showed that active expression of IMP1 suppressed the growth of MDA231 cell-derived breast tumors as well as pulmonary metastasis. Using microarray assays to analyze the profile of gene expression in IMP1-expressing or IMP1-nonexpressing tumors, we identified mRNAs that selectively associated with IMP1 and were post-transcriptionally regulated by the protein. Genetic manipulation of IMP1-bound mRNAs altered cell proliferation and invasive abilities, producing the same phenotypes as IMP1 knockdown. IMP1 truncate lacking the KH34 domain eliminated the RNA-binding activity of the protein, resulted in the loss of suppressive function for breast carcinoma cells and for tumor progression. In human breast tumors, IMP1 mRNA could be post-transcriptional regulated and its expression seemed to be correlated with the reduced levels of its associated transcripts, such as PTGS2, GDF15 and IGF-2 mRNAs. Our studies suggested a molecular mechanism that the ability of IMP1 to suppress breast tumor growth and metastasis could result from the regulation of its target mRNAs.

## RESULTS

### Orthotopic expression of IMP1 inhibits tumor growth in Scid/Scid mouse xenograft models

**Figure 1:**
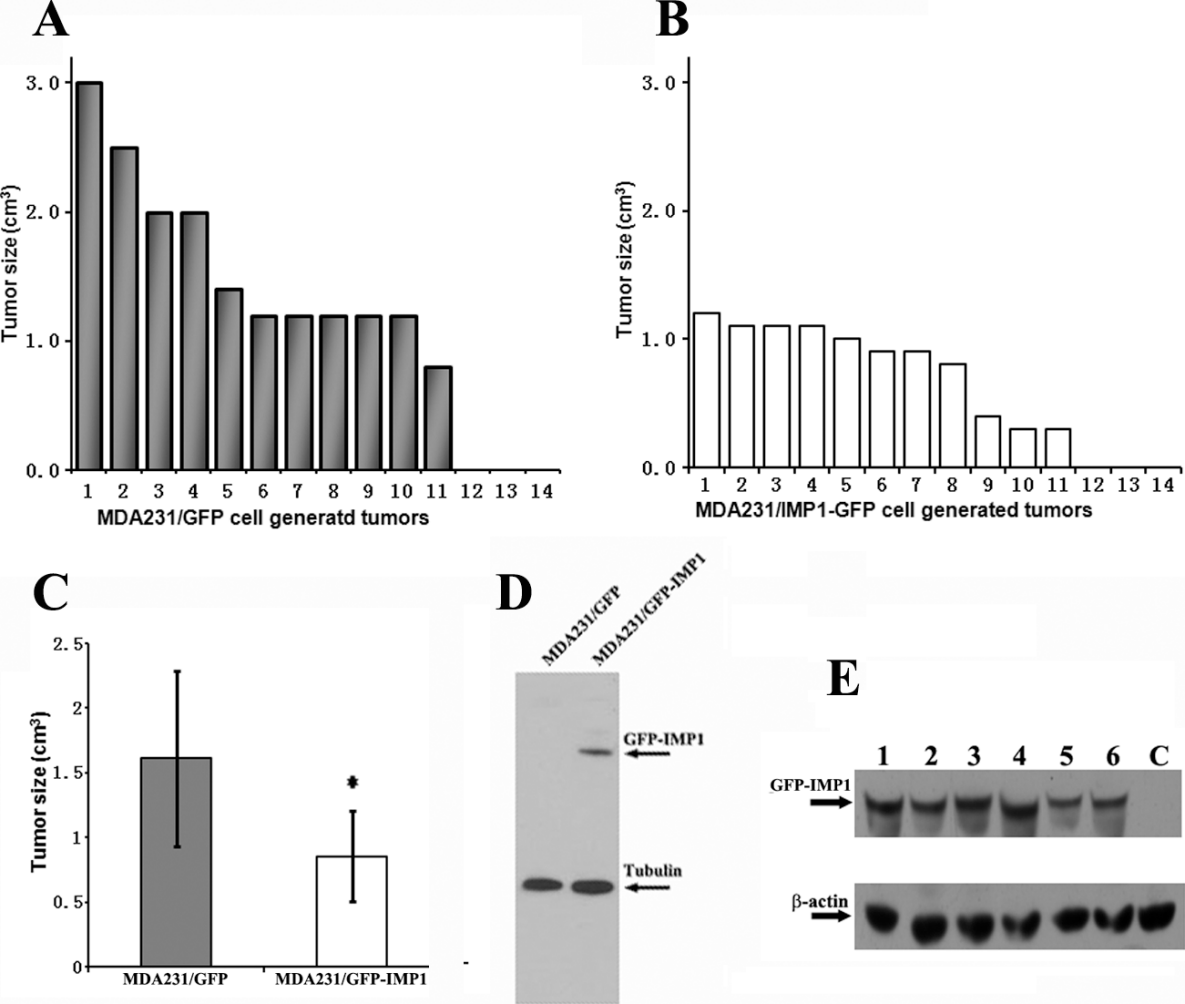
IMP1 expression inhibits the growth of MDA231 cell-derived breast tumors 11 of 14 mice injected with either MDA231/GFP or MDA231/GFP-IMP1 cells gave rise palpable tumors. The volumes of individual tumors derived from the two cell lines were indicated in (**A**) and (**B**). (**C**) Average tumor volumes in two xenograft groups were calculated in cm^3^. Bars indicate standard error of mean, *P* < 0.01. Tumors derived from MDA231/GFP-IMP1 cells displayed smaller primary tumor masses. (**D**) Immunoblots showing the expression of GFP-IMP1 in MDA231/GFP-IMP1 and MDA231/GFP cells. (**E**) Western blots were performed using antibody against IMP1 (Sigma). GFP-IMP1 was expressed in MDA231/GFP-IMP1 cell-derived xenografts (Lanes 1–6), but not in the xenograft derived from MDA231/GFP (lane C), β-actin antibody was used as a loading control.

Previous studies indicated that gain of IMP1 function in human metastatic MDA231 cells, which essentially lack IMP1 expression, increased cell polarity and attenuated invasive ability [7]. We hypothesized that the orthotopic expression of IMP1 would also disturb breast tumorigenesis and metastatic potential *in vivo*. To address this, we cultured MDA231/GFP-IMP1 cells that constitutively express Flag-tagged GFP-IMP1 and MDA231/GFP cells, and injected them into the mammary fat pads of Scid/Scid mice to generate xenograft tumors. Mice were sacrificed eight weeks postinjection, and the cell-derived breast tumors as well as the lungs were removed. Minor difference of total body weights between two groups was observed (not shown). 11 of 14 mice injected with either MDA231/GFP or MDA231/GFP-IMP1 cells gave rise palpable tumors. However, individual tumors derived from MDA231/GFP-IMP1 cells displayed smaller primary tumor masses (Figure 1A and 1B). A statistical analysis of the tumor volumes in two xenograft groups showed that the average volume of tumors that formed in mice injected with MDA231/GFP-IMP1 cells was about half of those injected with MDA231/GFP cells (Figure 1C, *P* < 0.01). Analyzing the expression of IMP1 by western blots confirmed the expression of GFP-IMP1 in MDA231/GFP-IMP1 cells (Figure 1D) and the cell-derived tumors (Figure 1E). No endogenous IMP1 was detected in the xenografts (not shown). These results indicated the inhibitory role of IMP1 for the xenograft tumor growth.

### Gain of IMP1 function suppresses lung metastases in Scid/Scid mouse xenograft models

**Figure 2:**
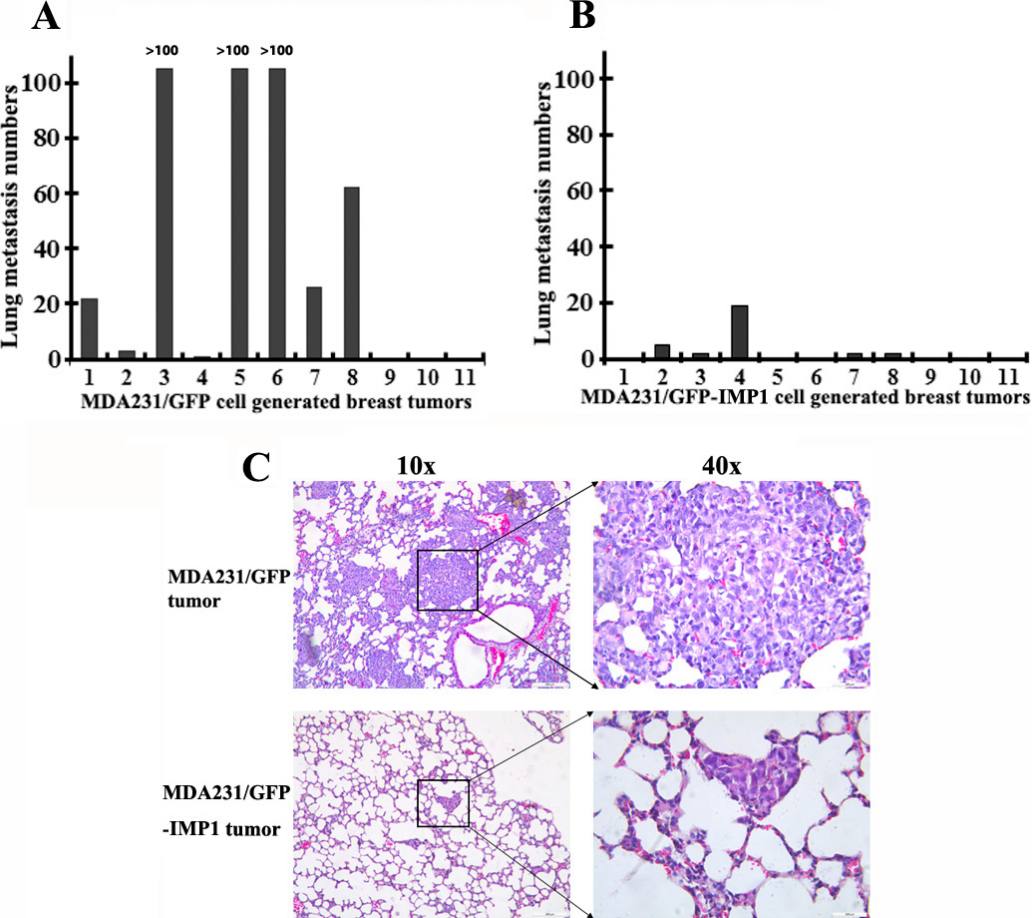
Gain of IMP1 function suppresses lung metastases (**A**) and (**B**) lung metastases were measured in individual mice with primary breast tumors derived from MDA231/GFP or MDA231/GFP-IMP1 cells. Numbers of lung metastasis in each individual mouse were measured. Rates of lung metastases were 73% in MDA231/GFP cell-derived tumors and 45% in MDA231/GFP-IMP1 cell-derived tumors (*P* = 0.0136). (**C**) Representative low-power and high-power H & E-stained, 5-um lung sections from mice injected with the indicated cells were shown. Visible metastatic nodules in the lungs of MDA231/GFP mice were markedly higher than those in the lungs of MDA231/GFP-IMP1 mice.

To examine the potential role of IMP1 in suppressing breast tumor metastasis, we dissected lungs from individual xenograft mice and used H & E staining to detect lung metastases. Results showed that 8 of 11 (~73%) MDA231/GFP cell-derived tumors, while 5 of 11 (~45%) MDA231/GFP-IMP1 cell-derived tumors developed lung metastasis (Figure 2A, 2B). Visual inspection indicated that MDA231/GFP tumor-metastasized lung nodules occupied a higher percentage of the total lung area, while IMP1-expressing tumor-metastasized lung nodules had discrete smaller dark foci. In addition, numbers of visible metastatic nodules in the lungs of MDA231/GFP mice were also markedly higher than those in MDA231/GFP-IMP1 mice (Figure 2C). The increased formation of lung metastases was not due to the increased growth of primary tumors, since the animal with largest volume of tumor did not give rise to higher lung metastases (Compare Figures 1A and 2A). These data suggest the suppressive effect of IMP1 in lung metastasis of breast tumors.

### Gene expression profiling of the xenograft tumors

Considering IMP1 is an mRNA regulator, the role of IMP1 to suppress breast tumor growth and metastasis could result from the regulation of its target mRNAs. To address this, we isolated total RNAs from four randomly selected tumors, two from MDA231/GFP-IMP1 and two from MDA231/GFP cell-derived xenografts, and performed microarray assays in Gene Company Limited in Shanghai, China (Data has been deposited in Gene Expression Omnibus as a submission number of GSE62638). The microarray results for the two MDA231/GFP-IMP1 cell-derived tumors or the two MDA231/GFP cell-derived tumors were highly similar. Based on the analyses provided by the company (*P* < 0.01), a total of 223 transcripts with at least a 2-fold change between MDA231/GFP-IMP1 and MDA231/GFP cell-derived xenografts were identified, in which 124 genes were up-regulated and 99 genes were down-regulated in responding to IMP1 expression (Supplementary Tables 2 and 3). Of particular interest in identifying the transcripts involved in breast tumor progression, some up-regulated transcripts functioning as tumor suppressors, including RGS4 (regulator of G-protein signaling 4), AMIGO2 (adhesion molecule with Ig-like domain 2) and RBP1 (Retinoic acid binding protein 1) mRNAs, and some down-regulated transcripts important for tumorigenesis, such as IGF-2, PTGS2 (prostaglandin-endoperoxide synthase 2) and GDF15 (growth differentiation factor 15) were selected and listed in Table S4A and Table S4B.

### Differential expression of microarray-identified transcripts in the xenograft tumors and in MDA231 cell lines

**Figure 3:**
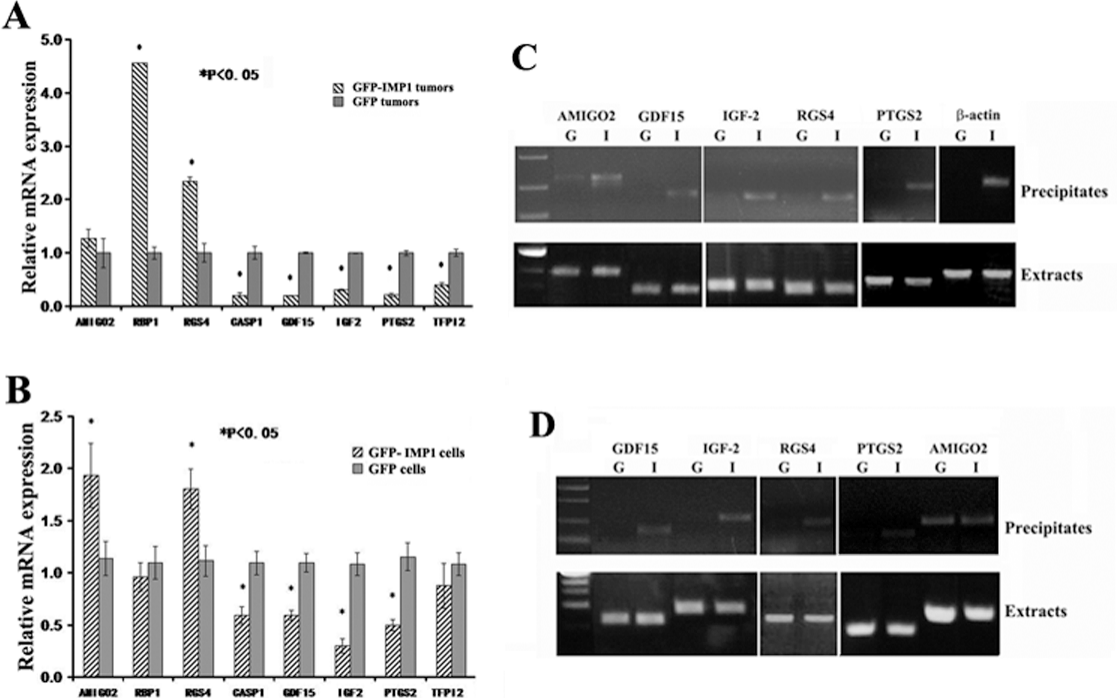
Differential expression of selected microarray-identified genes and their potential to bind to IMP1 Total RNA was extracted from the xenograft tumors (**A**) (*n* = 5, from each group) and from the cell lines used for generating xenografts (**B**). Real-time RT-qPCR was performed to detect the mRNA levels of eight selected genes identified in microarray assays. Relative expression levels of the mRNAs are statistically analyzed and the data are presented as means ± SEM from three independent experiments. *P < 0.05. (**C**) and (**D**) Immunoprecipitations were performed in the extracts prepared from MDA231 cell lines and from the xenograft tumors using anti-flag antibodies for flag-tagged GFP-IMP1. RNAs in the precipitates were extracted and used for RT-PCR assays. (C) Upper panels: GDF15, IGF-2, RGS4 and PTGS2 mRNAs were co-precipitated with IMP1 in the extracts of breast carcinoma cells, while AMIGO2 mRNA appeared to be non-specifically precipitated. β-actin mRNA was used as a positive control for IMP1 binding. G: MDA231/GFP cells; I: MDA231/GFP-IMP1 cells. Lower panels: RT-PCR indicated the presence of selected mRNAs in the extracts of carcinoma cells. (D) Upper panels: in addition to AMIGO2 mRNA that was non-specifically precipitated, GDF15, TFPI2, IGF-2 and PTGS2 mRNAs were also co-precipitated with IMP1 in the extracts of MDA231/GFP-IMP1 cell-derived xenograft tumors. Lower panels indicated the presence of selected mRNAs in the extracts of xenografts. G: MDA231/GFP cell-derived breast tumors; I: MDA231/GFP-IMP1 cell-derived breast tumors.

In order to confirm the microarray results that were truly indicated the differential pattern of gene expression in the xenograft animals, we examined expression of eight microarray-identified transcripts by real-time RT-PCR in randomly selected five individual tumors from MDA231/GFP-IMP1 or MDA231/GFP mice. These included three up-regulated genes: AMIGO2, RBP1 and RGS4, and five down-regulated genes: CASP1, GDF15, (IGF-2), PTGS2 or Cox-2 and TFPI2 (tissue factor pathway inhibitor 2). In consistent with the array results, statistic data indicated that the levels of AMIGO2, RBP1 and RGS4 mRNAs were up-regulated, and CASP-1, GDF15, IGF-2, PTGS2 and TFPI2 mRNAs were down-regulated the in individual xenografts expressing IMP1 (Figure 3A). Since the xenograft tumors were derived from MDA231/GFP-IMP1 or MDA231/GFP cells, we then examined the cellular expression of these selected transcripts to compare whether the differential expression was similar between carcinoma cell lines and the cell-derived tumors. Seven transcripts displayed a similar expression pattern as indicated in the tumor samples (Figure 3B). However, levels of RBP1 mRNA were slightly lower in IMP1-expressing cells than that in IMP1 non-expressing cells, indicating the expression difference of the gene between *in vivo* and *in vitro*.

### IMP1 physically interacts with microarray-identified transcripts in the xenograft tumors and in MDA231 cell lines

To determine whether IMP1 could physically directly interact with those selected microarray-identified transcripts *in vivo*, we performed IMP1 pull-down assays in the extracts of MDA231/GFP-IMP1, MDA231/GFP cells and of the cell-derived xenograft tumors, respectively, and used RT-PCR to analyze its associated mRNAs. Experiments showed that in addition to AMIGO2 mRNA that could be non-specifically precipitated, GDF15, IGF-2, PTGS2 and RGS4 mRNAs were identified only in the precipitates of IMP1-expressing MDA231 cells. As a positive control, β-actin mRNA was also co-precipitated with IMP1 (Figure 3C). No co-precipitation was detected for RBP1, TFPI2 and CASP1 mRNAs (not shown). We then analyzed whether IMP1 was also bound to selected transcripts in the xenograft tumors. Similarly, AMIGO2 mRNA was non-specifically precipitated, while GDF15, IGF-2, RGS4 and PTGS2 mRNAs were co-precipitated with IMP1 only in the extracts of IMP1-expression tumors (Figure 3D). No RNA degradation was observed in the extracts of cell and tumor samples (Lower panels of Figure 3C and 3D).

### IMP1 binds to the 3′UTR of GDF15 mRNA and regulates the translation of its bound mRNAs

**Figure 4:**
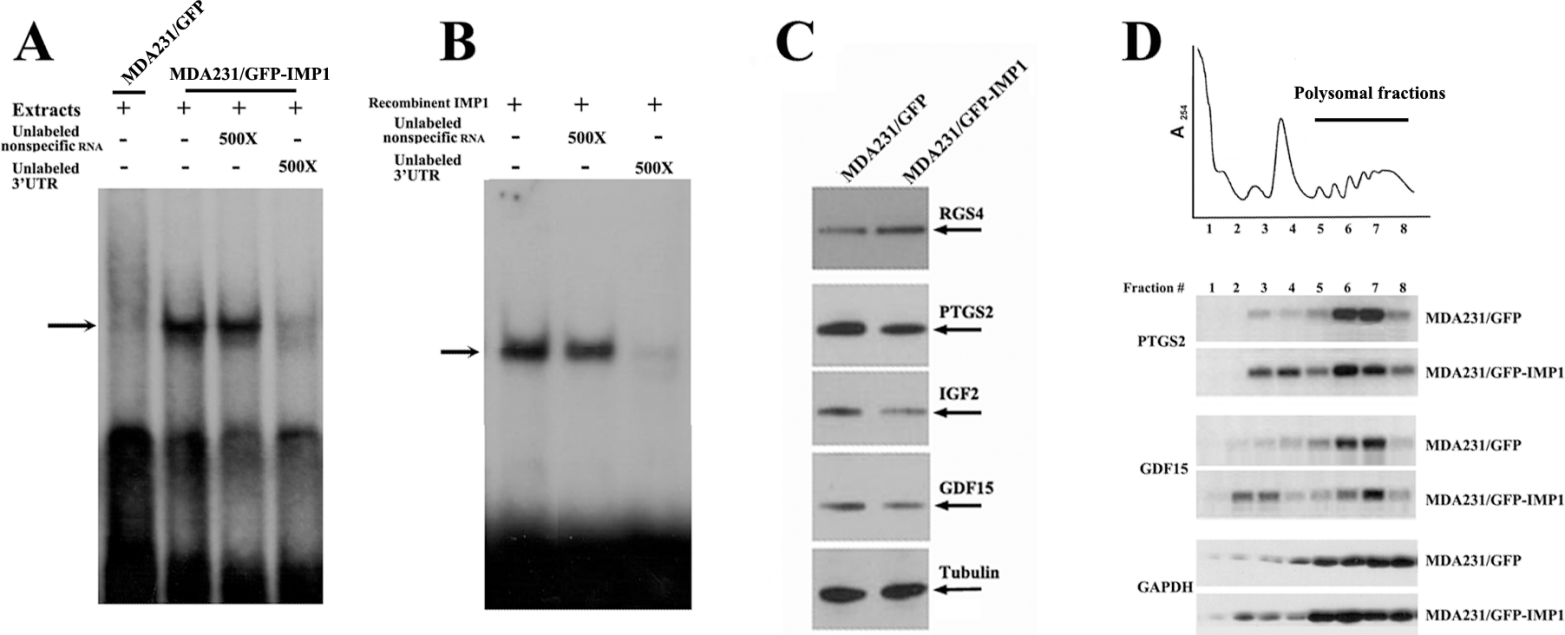
IMP1 binds to the 3′UTR of GDF15 mRNA and regulates the translation of its target mRNAs Aliquots of ^32^P-labeled 3′ UTR of GDF15 mRNA were incubated with extracts prepared from MDA231/GFP and MDA231/GFP-IMP1 cells (**A**) and recombinant IMP1 (**B**). RNA-protein complexes (arrow indicated) were formed when the RNA probe incubated with IMP1-containing extracts and with recombinant IMP1. Cell extracts without IMP1 expressing did not form complex with the RNA probe. The complexes were competed by 500× excess of unlabeled 3′ UTR of GDF15 mRNA, but not the non-specific RNA (yeast tRNA). (**C**) Western blots showing the protein expression of four IMP1-bound mRNAs in MDA231/GFP and MDA231/GFP-IMP1 cells. Tubulin was used as a loading control. (**D**) Extracts of MDA231/GFP-IMP1 and MDA231/GFP cells were fractionated in 10–50% linear sucrose gradients. An OD_254_ plot corresponding to the polysomal fractions was shown on the top panel. Total RNAs were isolated from the sucrose fractions. An equal amount of RNA from each fraction was subjected to Northern blots to detect the distribution of GDF15, PTGS2 and GAPDH mRNAs in the sucrose gradient.

Specific mRNAs are targeted for regulation by RNA binding factors that recognize sequences often found in the 3′ untranslated regions (UTR). To determine whether IMP1 was directly binds to its target mRNAs, we selected the 3′ UTR of GDF15 mRNA that contained an ‘ACACC’ motif previously identified for IMP1/ZBP1 recognition [19] and performed RNA gel-mobility shift assays. When radio-labeled 3′ UTR of GDF15 mRNA was incubated with the extracts of MDA231/GFP-IMP1 cells (Figure 4A) and purified recombinant IMP1 [20] (Figure 4B), a distinct RNA-protein complex was formed, (arrow indicated). The complex was able to be competed by 500× excess of unlabeled 3′ UTR of GDF15 mRNA but not non-specific RNA. No RNA-protein complex was formed when the extracts of MDA231/GFP cells were used (Figure 4A), indicating the complex was IMP1-specific.

To analyze whether IMP1 could affect the expression of its bound transcripts, we performed Western blots, which showed that, in consistent with the mRNA expression, levels of RGS4 protein was increased, and GDF15, PTGS2 and IGF2 proteins were reduced due to IMP1 expression (Figure 4C). To address the possibility that binding of IMP1 could repress the translation of its target mRNAs, we performed sucrose-gradient fractionations from MDA231/GFP and MDA231/GFP-IMP1 cells. An OD_254_ plot corresponding to the polysomal fractions was shown in Figure 4D. We isolated total RNA from the sucrose fractions and used Northern blots to analyze the polysomal distribution of PTGS2, GDF15 and GAPDH mRNAs (Figure 4E). GAPDH mRNA was mostly polysomal in both MDA231/GFP and MDA231/GFP-IMP1 cell lines (lower panel). Although PTGS2 and GDF15 mRNAs were mainly polysomal in MDA231/GFP cells (fractions 5–8), a substantial amounts of the transcripts were detected in non-polysomal fractions (fractions 2, 3, 4), indicating that not all the mRNA were translationally active in the presence of IMP1. We have also used RT-PCR to examine the polysomal profile of RGS4 mRNA, no changes were observed (Supplementary Figure 1). These results suggested that IMP1 could selectively suppresses the polysomal loading of its bound-mRNAs.

### The KH34 domain of IMP1 is responsible for mRNA binding and for IMP1 associated biological function

**Figure 5:**
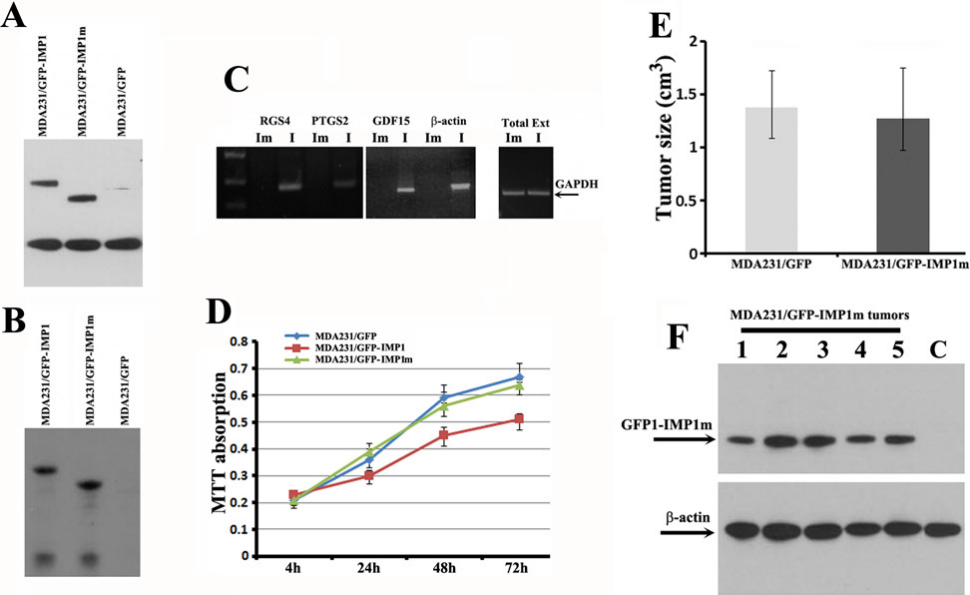
The KH34 domain of IMP1 is required for the target mRNA binding and for repressing proliferation of carcinoma cells as well as the growth of cell-derived breast tumors (**A**) Western blots showing the expression of IMP1 and IMP1 truncate in MDA231/GFP-IMP1 and MDA231/GFP-IMP1m lines. (**B**) Co-IP experiments were performed in the extracts prepared from MDA231 cell lines using antibodies against Flag-tag. Western blots indicated that the Flag-tagged GFP-IMP1 and GFP-IMP1m were successfully precipitated. (**C**) Co-precipitated RNAs were extracted and used for RT-PCR assays. GDF15, RGS4, PTGS2 and β-actin mRNAs were not precipitated when the KH34 domain of IMP1 was deleted. GAPDH mRNA was used as an internal control for the extracts used in the experiments. Im: MDA231/GFP-IMP1m cells. I: MDA231/GFP-IMP1 cells. (**D**) MTT assays were used to measure the rate of cell proliferation. Loss of RNA binding activity by deletion of KH34 domain abolished the ability of IMP1 in repressing proliferation of MDA231 cells (*P* < 0.005). Bars indicate standard error of mean from three independent experiments, (*P* < 0.01). (**E**) Average tumor volumes in MDA231/GFP and MDA231/GFP-IMP1m cell-derived xenograft animals, *P* > 0.5. (**F**) Western blots indicated the expression of GFP-IMP1m in MDA231/GFP-IMP1m cell-derived xenografts (Lanes 1–6). Lane C was a negative control for the xenograft derived from MDA231/GFP cells. Antibody for β-actin was used as a loading control.

We have previously shown that the KH34 domain of IMP1 is necessary for β-actin mRNA transport, so does the cell polarity and motility [20]. We hypothesized that binding of the IMP1 to the microarray-identified transcripts could also require the KH34 domain. To address this, we established a MDA231/GFP-IMP1m cell line, which stably expressed a Flag-tagged, KH34-deleted IMP1 truncation (GFP-IMP1m) (Figure 5A). We immunoprecipitated IMP1 and IMP1 truncate from the extracts of MDA231/GFP-IMP1 and MDA231/GFP-IMP1m cells (Figure 5B) and analyzed whether PTGS2, GDF15, IGF-2 and β-actin mRNAs could be co-precipitated with the proteins. All the four mRNAs were not precipitated in the extracts of MDA231/GFP-IMP1m cells (Figure 5C), indicating that the KH34 domain of IMP1 was necessary for interacting with the mRNAs. To further assess the ability of the KH34 domain of IMP1 for target mRNA binding, we used the 3′UTR of GDF15 mRNA to perform gel mobility shift assays using the extracts prepared from MDA231/GFP, MDA231/GFP-IMP1 and MDA231/GFP-IMP1m cells. When radio-labeled 3′ UTR of GDF15 mRNA was incubated with extracts of MDA231/GFP-IMP1 cells, the RNA-protein complex was detected (Supplementary Figure 2, arrow indicated). However, cell extracts expressing GFP or GFP-IMP1m did not form complex with the RNA probe.

Since IMP1 expression increased localization of β-actin and Arp-16 mRNAs at the cell leading edge thus maintained cell polarity [7], we postulated that IMP1 lacking the KH34 domain would impair their localization. FISH analysis indicated that, in contrast to MDA231/GFP-IMP1 cells, localization of β-actin and Arp-16 mRNAs was obviously reduced in MDA231/GFP-IMP1m cells (Supplementary Figure 3). To evaluate whether loss of the RNA binding ability would impair the role of IMP1 on cell proliferation and tumor progression, we performed MTT assays. Results showed that cells expressing the full-length IMP1 proliferated slower than IMP1 non-expressing cells. However, when the KH34 domain of IMP1 was deleted, the inhibitory effect of the protein to cell proliferation was also reduced (Figure 5D). We then made xenograft tumors by injecting the MDA231/GFP or MDA231/GFP-IMP1m cells into the mammary fat pads of Scid/Scid mice. 5 of 6 mice injected with MDA231/GFP cells, and 6 of 6 mice injected with MDA231/GFP-IMP1m cells gave rise palpable tumors. Analysis of the tumor volumes indicated that the average volume of tumors that formed in mice injected with either MDA231/GFP-IMP1m cells or with MDA231/GFP cells was not significantly changed (Figure 5E). Western blots indicated that GFP-IMP1m was truly expressed in MDA231/GFP-IMP1m tumors (Figure 5F) and no endogenous IMP1 was detected in xenografts (not shown). Thus, the ability of IMP1 to repress cell proliferation and tumor growth might be through the function of the KH34 domain.

### Knockdown of either IMP1 or RGS4 and PTGS2, the two IMP1-bound mRNAs, affects breast carcinoma cell proliferation and invasion

**Figure 6:**
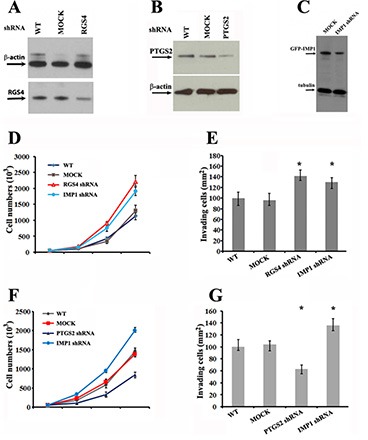
Knockdown of RGS4, PTGS2 and IMP1 expression affects proliferation and invasiveness of breast carcinoma cells (**A**), (**B**) and (**C**) Western blots showing the effects of shRNA on silencing RGS4, PTGS2 and IMP1 expression in MDA231 stable cell lines. (**D**) and (**F**) Effects of RGS4, PTGS2 and IMP1 knockdown on proliferation of MDA231 cells. Cell numbers were determined at the indicated time points. Data shown in the figure represent the means ± standard error of data from four independent experiments. (*P* < 0.01). (**E**) and (**G**) Knockdown of RGS4, PTGS2 and IMP1 expression changes the invasive potential of MDA231 cells. Cells were plated in serum-free medium into the upper chamber of 8 mm pore Matrigel-coated transwell filters. The lower chamber contained medium with 10% serum. Cells that had invaded to the underside of the filter were stained and counted in 16 hours. Relative numbers shown in the figure represent the means ± SEM. of data from three independent experiments, *P < 0.05.

Both of RGS4 and PTGS2 have been previously reported to involve in breast cancer cell proliferation and invasion [21, 22]. Based on the facts that IMP1 could regulate the expression of RGS4 and PTGS2 mRNAs, (Figures 4 and 5), we performed knockdown experiments to evaluate whether loss of IMP1 function could result in cell phenotypes as same as RGS4 and PTGS2 down-regulation. We used specific shRNAs to stably silence IMP1 or the two candidate genes separately in MDA231/GFP-IMP1 cells. Validation of shRNA silencing was conducted using western blots, which showed down-expression of the proteins in MDA231 cell clones (Figure 6A, 6B and 6C). MTT and transwell assays showed that, compared to mock cells expressing scrambled shRNA (Mock), either knockdown of RGS4 or IMP1 expression significantly increased the rate of cell proliferation and promoted cell invasion (Figure 6D and 6E). Opposite to IMP1 knockdown, down-regulation of PTGS2 markedly reduced the rate of cell proliferation and inhibited invasion (Figure 6F and 6G). Since RGS4 was up-regulated and PTGS2 was down-regulated in IMP1-expressing cells and xenografts, the data indicated that the function of IMP1 in suppressing tumor cell proliferation and invasion could through the regulation of its bound mRNAs.

### Correlation of IMP1 exprssion with the levels of its target transcripts in human breast tumors

**Figure 7:**
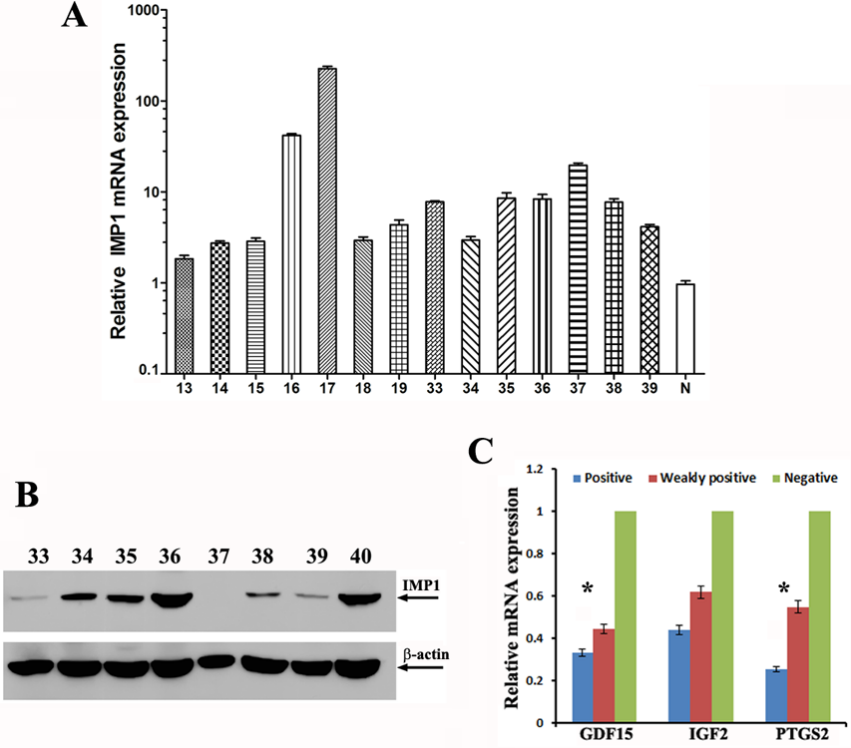
IMP1 expression correlated with the levels of its target mRNAs in human breast tumors (**A**) Relative levels of IMP1 mRNA in individual human breast tumors detected by RT-qPCR (Numbers are indicated as patient ID). Total RNA was extracted from 50 mg human breast tumors (*n* = 47) and breast normal tissues (*n* = 4), and 1 μg of total RNA was reversely transcribed. The resulting cDNA was analyzed by ABI 7500 Fast real time PCR system. Relative expression levels of IMP1 mRNA were calculated using comparative 2^−ΔΔ Ct^ method, and GAPDH was used as an endogenous control. Relative expression data was normalized to normal breast tissue (labeled as N). (*P* < 0.05). (**B**) Representative immunoblots indicate the expression of IMP1 protein in human breast tumors. Arrows indicate detected IMP1 and β-actin by their corresponding antibodies. (**C**) Relative levels of PTGS2, GDF15 and IGF-2 mRNAs in human breast tumors were reduced in response to increased IMP1 expression. Tumor samples were divided into three groups, one group is IMP1 negative (*N* = 15), the second one has lower levels of IMP1 expression (*n* = 11) and the third group expresses higher levels of IMP1 (*N* = 15). Total RNA was prepared from human samples and the levels of PTGS2, GDF15 and IGF-2 mRNAs were measured by qRT-PCR. Raw Ct values for each gene were normalized to raw Ct values for GPDH mRNA that was used as an internal control. Error bars represent standard deviation from three independent experiments. (*P < 0.01).

Previous studies have reported that IMP1 mRNA was expressed in about 58% of human breast patients [23]. However, using similar RT-PCR approach, we found that IMP1 mRNA was wildly expressed in human breast tumors (45/47, 96%) (Supplementary Figure 4A) and was even detected in some normal mammary tissues, (3/8, 38%) (Supplementary Figure 4B). We then used RT-qPCR to detect relative levels of IMP1 mRNA in individual tumor samples and found that the levels of IMP1 mRNA were largely variable among the samples tested (Figure 7A). In addition, levels of IMP1 mRNA in 13 of 14 tumor tissues were more than two folds of that in normal breast tissues. In comparison to mRNA expression, we used IMP1 antibodies to determine its protein expression. Immunoblots revealed that among 45 samples with detected IMP1 mRNA, only 31 samples (66%) showed protein expression. Interestingly, levels of the protein among tested patients were significantly different from their corresponding mRNA levels (Figure 7B), suggesting that the protein could be under translational control. No IMP1 protein was detected in patients who did not express the mRNA and in normal patient samples (not shown). Since the limited patient information, no connections between IMP1 levels and the pathological stages of the tumors were assessed. We next divided human breast tumor samples into three groups: IMP1 negative (*n* = 15), IMP1 weakly expressed (such as numbers 33 and 39 in Figure 7C, *n* = 11) and IMP1 highly expressed group (such as numbers 36 and 40 in Figure 7C, *n* = 21), and examined correlations between IMP1 expression and levels of PTGS2, GDF15 and IGF-2 mRNAs. Real-time RT-PCR indicated that IMP1 levels were proportionally related to the levels of GDF15, IGF2 and PTGS2 mRNAs. The highest levels of the transcripts appeared in the IMP1 negative group, while lower levels of the three mRNAs were in IMP1 highly expressed group (Figure 7C). Since the P values for IGF2 mRNA was not significant (*P* > 0.1), the results suggested a positive connection between increased levels of IMP1 and reduced expression of GDF15 and PTGS2 mRNAs in human breast tumors.

## DISCUSSION

We have previously reported that in breast cancer cells including MDA231, T47D and MTLn3 lines, loss of IMP1 function increased their proliferation and invasive potentials [7, 15]. Considering the *in vitro* studies may not reflect the complexity of tumorigenesis *in vivo*, we turned to utilize xenograft models to investigate the role of IMP1 in human breast tumor progression. In contrast to MDA231/GFP cell-derived xenografts, volumes of breast tumors derived from MDA231/GFP-IMP1 cells and lung metastasis were markedly decreased in a time course of 8 weeks. Thus, the xenograft models combined with *in vitro* cell-based studies provide insight into the positive role of IMP1 in repressing breast tumor growth and metastasis.

Several *in vivo* studies have indicated different actions of IMP1 in breast tumorigenesis [13, 16, 17]. For examples, targeted induction of IMP1 expression in mammary tissues of pregnant and lactating female mice developed breast adenocarcinomas that were capable of metastasizing [13]. In contrast, xenograft studies using two MTLn3 cell lines, one lacked IMP1 expression and the other expressing a chicken IMP1 homolog, demonstrated that active expression of IMP1 significantly repressed metastasis of the cell-derived breast tumors [16]. Our studies agreed with the inhibitory role of IMP1 in breast tumor growth and metastasis. The possible explanations for contradictive function of IMP1 could be from using different tumor models and be the complexity of breast tumorigenesis, indicating that tumor cells arrived from different origins or environmental conditions could behave differently in response to IMP1 expression. Interestingly, a recent study has reported that although IMP1 is involved in colorectal tumorigenesis, loss of IMP1 function in stromal cells provided a microenvironment that promoted colon tumor progression [18].

There are many other proteins having opposite biological functions in different cancer types. For examples, expression of NAG1/GDF15 in esophageal squamous cell carcinomas was significantly correlated with several malignant phenotypes including vessel invasion and lynph node metastasis [24]. However, a role of anti-tumorigenesis for the protein in transgenic mice was also reported [25]. Another example is EGR-1 that has been shown to promote tumorigenesis of prostate cancer [26], whereas it also indicated the tumor-suppressive effect [27]. In addition, LOX-1 acts either as a pro-tumorigenic protein in prostate cancer or as a tumor suppressor in colorectal cancer [28, 29]. Thus, it might be not surprising that IMP1 shows the dual functions in carcinogenesis.

Wang et al have previously demonstrated a number of transcripts that were up- or down-regulated following IMP1 expression in MTLn3 cell-derived rat xenografts [16], however, how IMP1 affected metastasis by regulating its target transcripts was not investigated. In this study, we revealed handful IMP1-bound transcripts whose expression was correlated to IMP1 expression. These transcripts included RGS4, IGF-2, GDF15 and PTGS2 mRNAs. Ample studies have revealed the biological roles of these genes. For examples, increased expression of RGS4 mRNA inhibited breast cancer cell migration and invasion [22]. GDF15 was implicated in multiple cancer types and correlates with lymph node metastases in endometrial cancer [30, 31] and overexpression of IGF-2 or PTGS2 mRNAs in breast tumors associated with greater mortality and increased metastatic potential [32, 33]. Since IMP1 physically bound to these mRNA in breast tumor cells, we hypothesized that the effects of IMP1 in suppressing xenograft tumor progression might be partly attributed to the interaction and inhibition of these tumor cell growth and metastasis-related transcripts. This hypothesis was addressed by the experiments that increasing the expression of either GDF15 or PTGS2 mRNA in breast cancer cells generated same phenotype as IMP1 knockdown.

Our data demonstrated that binding of IMP1 to the transcripts identified in the study required the KH34 domain. One of the examples is that the 3′UTR of GDF15 mRNA only binds to full-length IMP1, but not the IMP1 truncate without the KH34 domain. Interestingly, the 3′UTR of GDF15 mRNA contains an ‘ACACCC’ sequence, which was previously identified as IMP1 binding motif [19]. IMP1 lacking the KH34 domain completely abolished its function in inhibiting proliferation and migration behavior of breast cancer cells, most likely resulted in the loss of the binding ability to its target mRNAs. This is consistent with the studies by Oberman et al. that deletion of the KH34 domain of IMP1 (VICKZ) affected the cell migration capability [34]. Interestingly, searching the IMP1-bound mRNAs for the consensus sequences predicted for the KH34 domain binding [35] did not identify matched sequences, suggesting that binding of IMP1 to these transcripts could be through other structural constrains.

Previous studies reported that the IMP1 gene has been activated in breast tumors (58.5%), but not in normal tissues of adult origin [23]. Using the same approach, we detected IMP1 mRNA that was widely expressed in human breast carcinomas (96%) as well as in non-carcinoma breast tissues (38%). Surprisingly, among the tumor samples with detectable expression of IMP1 mRNA, many showed no corresponding protein expression, implying that IMP1 mRNA in those tumors could be translationally repressed. In addition, higher IMP1 protein levels correlated with lower expression of GDF15, PTGS2 and IGF2 mRNAs in human breast tumors. Due to the relative small numbers of samples used and limited clinical information, we are unable to statistically determine the correlations of the levels IMP1 protein with tumor stages or with the metastatic status of the patients. Therefore, to investigate the underlying mechanism of post-transcriptional regulation of the IMP1 gene and the physiological role of IMP1-mediated transcripts in human breast tumor progression and metastasis will be the focuses of our future studies.

In conclusion, our studies indicated a suppressive role of IMP1 in breast tumor growth and metastasis. This study is consistent with our previous *in vitro* results that IMP1 could repress proliferation and invasiveness of breast carcinoma cells [7, 36]. The ability of IMP1 to suppress breast cancer progression could result from the regulation of its target transcripts, leading to changes in cancer cell behavior including proliferation and invasiveness.

## MATERIALS AND METHODS

### Xenograft tumor mice

MDA231/GFP-IMP1 and MDA231/GFP cell lines were generated by stable infection of lentivirus expressing flag-tagged IMP1-GFP or GFP in parental ATCC line, as described previously [7]. All procedures used for generating cell-derived xenografts were conducted in accordance with the National Institutes of Health regulations and approved by Animal Care and Use Committee in the Shantou University Medical College. Cultured cells were resuspended in DMEM media (4 × 10^7^/ml) and mixed with Matrigel (BD Bioscience) at 1:1 ratio. 200 μl of cell mixture were injected with a 22-gauge needle into the fat pad of mammary gland of anesthesized 6-wk-old Scid/Scid mice (BeiJing Animal Facility Center, China). Animals were kept at four mice per cage in microisolator units and provided with sterile water and chow for 8 weeks. Mammary tumors were collected and stored at −80c freezer after measuring the tumor volumes in cubic millimeter by using a 2 ml cylinder. Lungs were removed and fixed in 10% formalin, and embedded in paraffin.

### Measurement of lung metastases

For measurement of metastases, excised lungs were placed in 3.7% formaldehyde, mounted in paraffin, sectioned, and stained with hematoxylin and Eosin (H & E). Slices were viewed using a 20× objective and counted the metastatic lesions in each section. All of the metastases in a section containing 5 or more cells were counted.

### Primary human tissue samples

All human breast tumor tissue was received as discarded tissue (that is, excess tumor tissue after enough specimen was collected by the Shantou Tumor Hospital Pathology Department for diagnostic tests). Because the tissue was not collected specifically for the proposed study and did not contain a code derived from individual personal information, no patient consent was required. Tumor tissue was assigned a random number ID when received at the laboratory. Adjacent non-neoplastic tissues at the proximal surgical region were taken in the course of direct surgery. The experiments were approved by the Shantou Tumor Hospital (Chairman, Professor GuoJun Zhang), and operated according to International Conference on Harmonisation (ICH) / WHO GCP and the applicable laws and regulations.

### Stable cell lines and cell culture

Flag-tagged IMP1 truncate (IMP1m, aa 1–404) that lacks the KH34 domain was PCR amplified and subcloned into a lentiviral vector downstream of the GFP gene. shRNA vectors for RGS4 and PTGS2 mRNAs were purchased from OriGene Technologies (TR30007 and TG310074). The vectors were used to infect MDA231 cells. MDA231 cells infected with the above vectors were seeded in a six-well dish at 20% confluence, as previously described [15]. Stably infected cell clones were separated by FACS according to their green fluorescence intensities were then cultured at 37°C in a humidified 5% CO2 incubator in Dulbecco's modified Eagle's medium (DMEM) supplemented with 10% fetal bovine serum (FBS).

### Total RNA and protein extraction

Extraction of total RNA from MDA231 cells were carried out as previously described [37]. To prepare total RNA and protein extracts from xenograft tumor and from human tissues, aliquots of tumor samples were grounded in 10 mM Tris-HCl buffer, pH 7.4, containing protease inhibitors (Roche) and RNase inhibitor (Invitrogen). Supernatants were obtained after high-speed centrifugation. Part of the supernatants was stored in liquid nitrogen for protein analysis. Totoal RNAs were extracted from the supernatants using TRIzol as described by the manufacturer (Invitrogen).

### Microarray experiments and data deposit

Microarray experiments and data analyses were performed at the Gene Company Limited in Shanghai, China. The microarray data has been deposited in Gene Expression Omnibus (GEO) as a submission number of GSE62638.

### RT-PCR and real-time qRT-PCR

First-strand cDNA synthesis was performed using approximately 1 μg of total RNA, oligo dT primers and Superscript III RNase reverse transcriptase (Invitrogen). Synthesized cDNA was used for PCR amplification with the specific primers listed in Supplementary Table 1. All PCRs were carried out in a S1000^TM^ thermal cycler (Bio-Rad) with the following parameters: [30–35] cycles of 30 sec at 95°C, 30 sec at 55°C and 30 sec at 72°C. Quantitative reverse-transcription-PCR (qRT-PCR) was performed to analyze transcripts identified by microarray assays. The SYBR Green PCR Core Reagents system (Applied Biosystems, Foster City, CA) was used for real-time monitoring of amplification.

### SDS-PAGE and immunoblotting analyses

Cell lysates were mixed with SDS loading buffer and boiled for 5 min before cooling on ice. Samples were loaded on a 4–12% gradient gel (Invitrogen) and subjected to electrophoresis. Proteins were then transferred to nitrocellulose membrane and subjected to immunoblotting analysis. Primary antibodies used for the blots include: mouse monoclonal antibodies against IMP1, PTGS2 and Flag-tag, hamster monoclonal anti-RGS4 antibodies (Santa Cruz, Dallas, TX), and mouse anti-β-Actin monoclonal antibodies (Sigma-Aldrich, St. Louis, MO).

### Sucrose-gradient fractionation and northern blots

Cells were cultured in regular to DMEM medium containing 10% FBS and were harvested. Cell lysates were centrifuged for 30 minutes at 21,000 g and the supernatants were loaded onto 10 ml, 10–50% linear sucrose gradients and fractionated at 35,000 r.p.m. for 2 hours in an SW41 rotor (Beckman). Fractions (1.25 ml each) were collected from top to bottom and the OD_254_ profile was monitored. For Northern blots, total RNA was extracted from the sucrose tractions and equal amounts of total RNA were separated by agarose gel electrophoresis. RNA was transferred to a Hybond membrane and incubated with ^32^P labeled cDNA probes for GDF15, PTGS2 and GAPDH mRNAs. Hybridization was performed as previously mentioned [37].

### Gel mobility-shift assay

A 240 bp cDNA fragment encoding the 3′ UTR of GDF15 mRNA was PCR amplified and subcloned into pSP64 plasmid (Promega). ^32^P-labeled RNA probes were *in vitro* generated by SP6 RNA polymerase from pSP64-GDF15 3′ UTR construct. Transcribed RNA probes were purified after resolving in a 6% denaturing gel. RNA–protein gel-shift assays were performed at room temperature as described previously. The RNA–protein complexes formed were separated by electrophoresis in a 4% native gel and visualized by autoradiography.

### Isolation of IMP1 mRNP complexes and RNA extraction

Briefly, MDA231/GFP, MDA231/GFP-IMP1 and MDA231/GFP-IMP1m cells were lysed in an ice-cold lysis buffer containing 10 mM HEPES, pH 7.8, 40 mM NaCl, 10 mM KCl, 0.5% NP-40, 0.5 μg/ml PMSF, and 1× protease inhibitor mixture (Roche). Tumor samples were suspended into the lysis buffer and grounded in a homogenizer. Supernatants were obtained after high-speed centrifugation (21,000 g for 60 minutes at 4°C) and were incubated with FLAG-specific monoclonal antibody M2 covalently coupled agarose beads (Sigma) at 4°C in the presence of RNase inhibitor (250 units/ml). After overnight incubation, the supernatant was removed by a short centrifugation and the beads were extensively washed in lysis buffer followed by adding 1 ml of TRIzol. RNAs were extracted in TRIzol as described by the manufacturer (Invitrogen). Aliquots of total RNA was used to detect the expression of genes interested.

### Cell proliferation assay

To test the role of RGS4 and PTGS2 on cell proliferation, equal amounts of MDA231 and MDA231/RGS4-shRNA and PTGS2-shRNA cells were seeded and cultured for [24, 48, 72] and 96 hours. Cell counts were obtained by standard trypan blue (Sigma) staining. The BrdU-incorporation assay was performed using the BrdU Cell Proliferation Assay Kit following the manufacturer's instruction (Calbiochem). A total of 20 μl of BrdU-labeling reagent was added to each well of a 96-well culture plate containing tested cells in 100 μl (0.5 × 10^5^/ml) of culture medium. Cells were incubated for periods of [12, 24] and 36 hours at 37°C. The absorbance at 495 nm was measured using a 96-well plate reader (Tecan).

### Cell invasion assays

Cell invasion experiments were performed using BD BioCoat growth factor reduced Matrigel invasion chambers according to the manufacturer's protocol (BD Biosciences). Briefly, 200 μl MDA231 cells (1 × 10^5^) suspended in DMEM medium containing 0.5% BSA was added to the upper chamber and DMEM medium containing 10% FBS was added to the lower chamber. Cells were allowed to invade through the Matrigel for 24 hours. The invasive cells underneath the chamber were fixed in 3.7% formaldehyde in phosphate-buffered saline (PBS) for 15 minutes and stained with 0.2% crystal violet in 2% ethanol for 10 minutes. Noninvasive cells were scraped from the top chambers. The level of invasion was quantified by visual counting of the cells on the underside of the membrane. Each experiment was performed three times, and the results were expressed as means + s.e.m.

## SUPPLEMENTARY MATERIALS FIGURES AND TABLES






