# Supplementary material for: IMP1 suppresses breast tumor growth and metastasis through the regulation of its target mRNAs
Source: Oncotarget. 2016 Feb 17;7(13):15690–702. doi: 10.18632/oncotarget.7464 (PMC4941270; doi:10.18632/oncotarget.7464)
Supplement: Supplementary file 1 [file oncotarget-07-15690-s001.pdf]

IMP1 suppresses breast tumor growth and metastasis through the regulation of its target mRNAs

Supplementary Materials

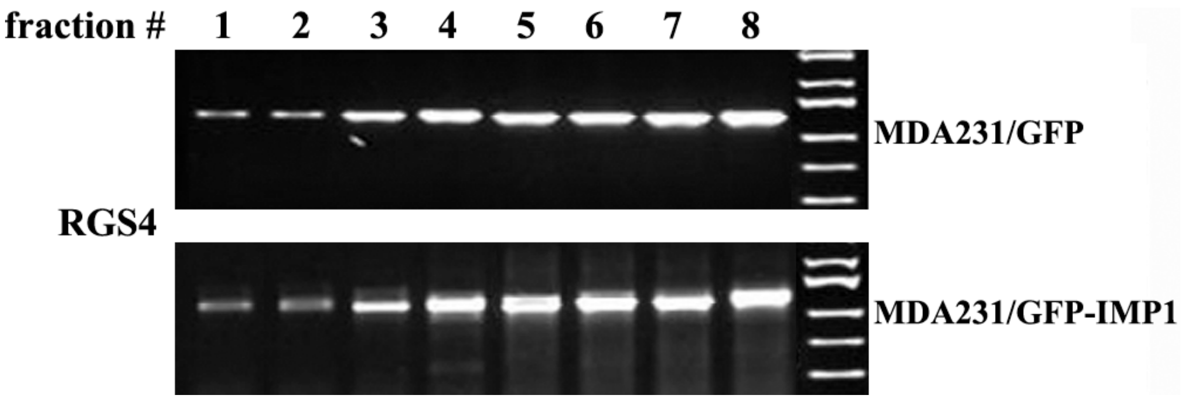

Supplementary Figure S1: Total RNAs were isolated from the sucrose fractions. An equal amount of RNA from each fraction was subjected to RT-PCR to detect the polysomal distribution of RGS4 mRNA in the sucrose gradient.

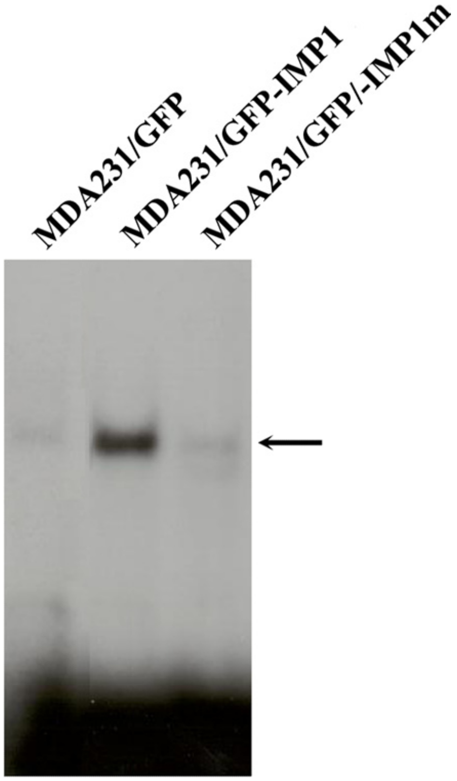

Supplementary Figure S2: The 3′UTR of GDF15 binds to IMP1 but not to IMP1m. Aliquots of ^{32}P-labeled 3′ UTR of GDF15 mRNA was incubated with extracts prepared from MDA231/GFP, MDA231/GFP-IMP1 and MDA231/GFP-IMP1m cells. RNA-protein complexes (arrow indicated) were formed in the MDA231/GFP-IMP1 extracts. Cell extracts expressing GFP or GFP-IMP1m did not form complex with the RNA probe.

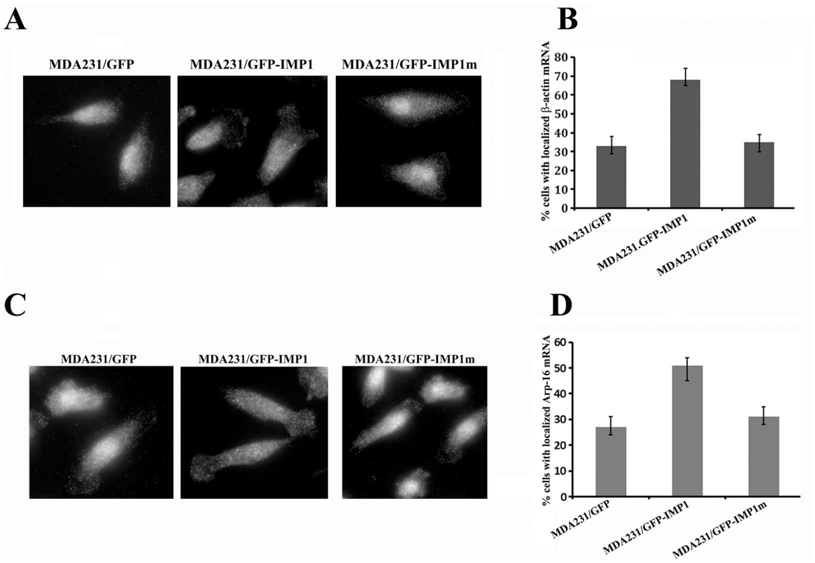

Supplementary Figure S3: Localization of β-actin and ARP-16 mRNAs in MDA231 cells in responding to IMP1 or IMP1m expression. *In situ* hybridization was performed on MDA231/GFP, MDA231/GFP-IMP1 and MDA231/GFP-IMP1m) cells to detect the localization of β-actin mRNA (**A**) and (**B**), and ARP-16 mRNA (**C**) and (**D**). (n = 100 cells from each cell clone, from two independent experiments). Error bars indicate ± s.e.m.

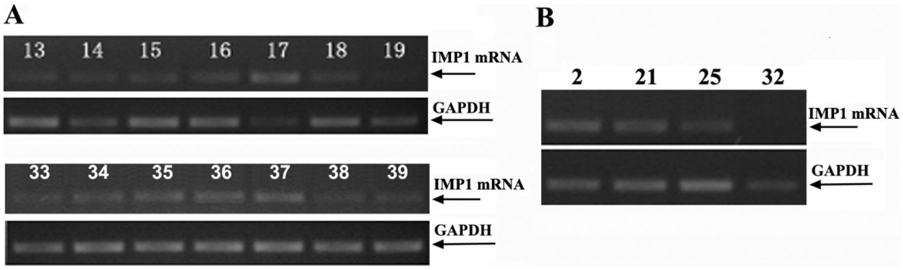

Supplementary Figure S4: Expression of IMP1 mRNA in human breast tumor and normal tissues. (**A**) Representative agarose gels showing the expression of IMP1 mRNA in human breast tumors. (**B**) Representative agarose gels showing the expression of IMP1 mRNA in normal breast tissues. Tissue samples were obtained from Shantou Tumor Hospital, China. Transcripts of IMP1 were detected by nested RT-PCR. GAPDH mRNA was used as internal controls for the integrity of RNA. Arrows indicate the amplified IMP1 and GAPDH mRNAs.

Supplementary Table S1: Primers used for RT-PCR

Gene	Forward primers	Reverse primers
AMIGO2	ATGTACTGGATAATGCCACATCC	CAAAGCACAGTACAGTAGGGTC
CAPS1	AATGGAGGCTGCAGGACTGGC	AGTCCGGGTGCGGGGATCGG
CDH5	TACACCTCGCTGTTGTCACATC	ATGACGAAGGGTGAGCTTGGTG
CEBPB	CAAAACTTTGGCACTGGGGCAC	TTGCGTCAGTCCCGTGTACACA
GAPDH	GAGTCAACGGATTTGGTCGT	TGGGATTTCCATTGATGACA
GDF15	GGTGCTCATTCAAAAGACCGAC	ACAGCTGTTTGGGCAGGAATCG
IGF2	CCAATATGACACCTGGAAGCAG	ACTTCCGATTGCTGGCCATCTC
IMP1	CAGCCAGCTGACTTCAGTCACC	ACGTGCTTTTACATAGCAGTGGC
Kiss1	ACTCACTGGTTTCTTGGCAGC	ACCTTTTCTAATGGCTCCCCA
PTGS2	GCCTGAATGTGCCATAAGACTG	TATTTCATTTCTCCCTCTTCCC
RBP1	CCATATGATCATCCGCACGCTG	ACTGCACCTTCTTGAATACTTGC
RGS4	AAGATGTGCAAAGGGCTTGCAG	AGCCCATTTCTTGACTTCCTC
TFPI2	GTCCCAAGAAGACAAAGTCGCA	GTGGTCTCCAACCCACAATGTC
TNFAIP2	CTGACACCATCCAGCACTTCTG	GACCTCACTGTTGGATAGGTTC

Microarray identified transcripts were selected for further validation by real time RT-PCR. Primers listed in the table were used to detect the expression of interested genes in xenograft mice, MDA231 cells and human patients.

Supplementary Table S2: Genes are up-regulated in xenograft tumors in responding to IMP1 expression

Microarray identified mRNAs that are up-regulated in the xenograft tumors in responding to IMP1 expression (I/G). Microarray experiments and data analyses ($P < 0.05$) were performed in the Gene Company Limited (GuangZhou, China). Transcripts that were up-regulated in responding to IMP1 expression were shown. I: GFP-IMP1 cell-derived tumors; G: GFP cell-derived tumors.

Supplementary Table S3: Genes are down_regulated in xenograft tumors in responding to IMP1 expression

Microarray identified mRNAs that are down-regulated in the xenograft tumors in responding to IMP1 expression (I/G). Microarray experiments and data analyses ($P < 0.05$) were performed in the Gene Company Limited (GuangZhou, China). Transcripts that were down-regulated in responding to IMP1 expression were shown. I: GFP-IMP1 cell-derived tumors; G: GFP cell-derived tumors.

Supplementary Table S4: Selected microarray-identified transcripts up- or down-regulated in xenograft tumors

A. Selected mRNAs up-regulated in xenograft tumors

Gene symbol	Gene bank ID	Scald fold GFP-IMP1/GFP	Biological functions
ZDHHC17	NM_015336	2.0	Cellular transport and signal transduction
ARHGAP11A	NM_199357	2.1	GTPase activator, small gtpase mediated signal transduction
MAPK14	NM_139012	2.5	Signal transduction and apoptotic process
TNS1	NM_022648	2.9	Fibroblast migration, cell-substrate junction assembly
ARHGAP26	NM_001135608	3.0	GTPase activator, actin cytoskeleton organization
AMIGO2	NM_001143668	3.7	Negative regulation of apoptotic process and cell adhesion
TAGLN	NM_001001522	5.1	Epithelial cell differentiation, muscle organ development
CDH5	NM_001795	5.4	Negative regulation of cell proliferation, cell polarity
GNGT2	NM_001198756	5.5	Signal transducer and phototransduction
RGS4	NM_001113380	5.6	Regulation of G-protein coupled receptor protein signaling pathway
CD24	NM_013230	7.0	Wnt signaling pathway
RBP1	NM_001130992	7.0	Retinoid metabolic process
PTPRN2	NM_130842	7.8	Transmembrane receptor protein tyrosine phosphatase
KISS1	NM_002256	10.3	Repressing cell proliferation, Regulation of MAPK cascade

B. Selected mRNAs down-regulated in xenograft tumors

Gene symbol	Gene bank ID	Scald fold GFP-IMP1/GFP	Biological functions
MUC1	NM_001044390	0.46	Transcription cofactor, post-translational protein modification
PPP1R3C	NM_005398	0.41	Protein serine/threonine phosphatase
CDH18	NM_001167667	0.38	Calcium ion binding, adherens junction organization
CEBPB	NM_005194	0.38	Cell proliferation, cell differentiation and immune response
GDF15	NM_004864	0.36	Cell proliferation, extracellular matrix organization
ZFP36	NM_003407	0.36	RNA metabolic process, AU-rich element binding
TNFAIP2	NM_006291	0.35	Angiogenesis, cell differentiation, exocytosis
RASD1	NM_016084	0.34	Signal transduction and regulation of transcription
BATF	NM_006399	0.25	Cell differentiation, cytokine production, transcription
TFPI2	NM_006528	0.17	Extracellular matrix constituent, serine-type endopeptidase inhibitor
CEBPD	NM_005195	0.15	Sequence-specific DNA binding
CASP1	NM_033295	0.15	Signal transduction, innate immune response
GPR64	NM_001079858	0.07	G-protein coupled receptor activity
RAP1A)	NM_002884	0.03	Ras GTPase binding, regulation of insulin secretion
PTGS2	NM_000963	0.03	Peroxidase activity and protein homodimerization activity

Selected microarray identified mRNAs that are up- or down-regulated in the xenograft tumors (IMP1+/IMP1−). Two categories of transcripts important for tumorigenesis and metastasis were selected from the microarray results. One category of mRNAs contained transcripts that were up-regulated in the presence of IMP1 (**A**). The second category included those down-regulated mRNAs in IMP1-expressing tumors (**B**).
